# Thermal Control of Concentric Topographies Patterned by Dynamic Electro‐Templated Generated Chiral Solitonic Structures

**DOI:** 10.1002/advs.76885

**Published:** 2026-07-29

**Authors:** Jacques A. Peixoto, Hanqing Zhao, Dirk J. Broer, Ivan I. Smalyukh, Danqing Liu

**Affiliations:** ^1^ Laboratory of Human Interactive Materials (HIM), Department of Chemical Engineering and Chemistry, Eindhoven University of Technology Eindhoven AZ The Netherlands; ^2^ Institute for Complex Molecular Systems (ICMS) Eindhoven University of Technology Eindhoven MB The Netherlands; ^3^ Department of Physics University of Colorado Boulder Colorado USA; ^4^ Department of Electrical, Computer, and Energy Engineering, Materials Science and Engineering Program University of Colorado Boulder Colorado USA; ^5^ Renewable and Sustainable Energy Institute, National Renewable Energy Laboratory University of Colorado Boulder Colorado USA; ^6^ International Institute for Sustainability with Knotted Chiral Meta Matter (WPI‐SKCM2) Hiroshima University 1‐3‐1 Kagamiyama Higashi‐Hiroshima Hiroshima Japan

**Keywords:** chirality, liquid crystal networks, self‐assemblies, stimuli‐responsive materials, surface modulations, topological solitons

## Abstract

Topological solitons in chiral nematic liquid crystals offer an elegant route to program complex director fields and dynamic surface responses. However, controlling these structures with spatial precision and scalability remains a key challenge. Here, an electro‐templating strategy is introduced to control the relaxation of cholesteric finger loops, enabling the generation of discrete, concentric solitonic ring structures with programmable geometry. This process exploits the structural multistability stemming from the interplay between the tendency to twist at a rate of helical pitch and confinement, allowing for on‐demand formation of multi‐ring architectures governed by relaxation dynamics in response to pre‐designed voltage driving. Upon photopolymerization, the resulting polymer retains the topological configuration of the soliton and exhibits thermally induced, reversible surface modulation, forming castle‐like structures with a central pillar and tunable concentric walls. Supported by theoretical modeling, this approach offers a scalable platform for encoding highly resolved hierarchical topological features into soft materials, opening new opportunities for responsive coatings and adaptive optics.

## Graphical abstract

An electro‐templating strategy directs the relaxation of cholesteric finger loops into discrete concentric rings whose geometry is electrically programmed. The photopolymerized structures retain the topological defect pattern and, upon heating, show reversible, castle‐like surface modulation. This scalable platform encodes hierarchical topological features into soft coatings for soft robotics, adaptive optics, and anti‐counterfeiting applications.

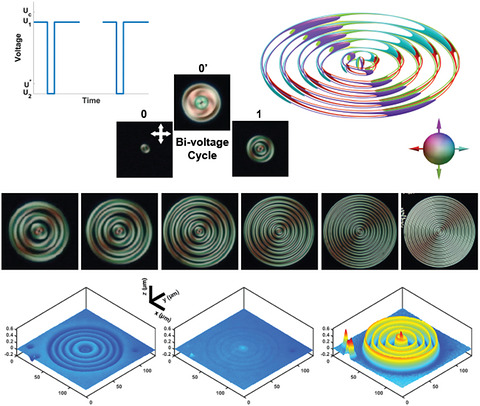

## 1 Introduction

Adaptive materials capable of encoding structural complexity and dynamic responsiveness are central to the development of next‐generation soft technologies, including haptic interfaces, reconfigurable optics, and smart microfluidics [1, 2, 3]. Inspired by natural systems, such materials harness external stimuli (such as temperature, light, or mechanical force) [4, 5, 6] to achieve precise, reversible transformations in shape, surface topography, and optical properties.

Among these materials, liquid crystalline systems offer a unique convergence of molecular order and mesoscopic responsiveness, supported by their anisotropic elasticity and rich structural and phase behavior [7, 8, 9]. In particular, chiral nematic (cholesteric) phases provide fertile ground for realizing topological solitons (self‐assembled localized structures such as torons [10], skyrmions [9], hopfion [10], and cholesteric fingers) [11] that arise from the interplay between helicoidal twist and confinement geometry [11, 12]. These solitons not only serve as model systems for theoretical purposes [13, 14] but also hold promise for photonics [15, 16, 17, 18, 19], and soft robotics [20, 21, 22].

Here, we introduce a scalable, electro‐templating strategy to program complex, spatially resolved topological architectures in a chiral nematic medium. By leveraging the metastable transformation of cholesteric finger 1 loops (CF1Ls) into torons [9, 23, 24], we control the formation of concentric ring‐shaped solitonic structures with a tunable number of closed loops (*m*). This transformation is driven by controlled relaxation dynamics and geometric confinement, enabling on‐demand fabrication of multiring solitonic configurations far exceeding the original feature size.

Upon photopolymerization, the cholesteric textures are locked into a solid polymer network, preserving their characteristic localized director field and topological structure. The resulting materials in the form of films embedding such solitonic structures can display a distinctive “castle‐like” surface morphology, with a central pillar surrounded by concentric polymer walls that respond reversibly to thermal stimuli. Supported by a numerical model combining the energetics of the liquid crystal solitons before polymerization with the response to external stimuli after polymerization, our approach provides a pathway toward lithography‐free surface patterning of soft materials with tunable optical and mechanical responses, advancing the design of adaptive functional interfaces.

## 2 Results and Discussion

### 2.1 Unwinding the Pitch by the Mean of an Applied Electric Field

**FIGURE 1:**
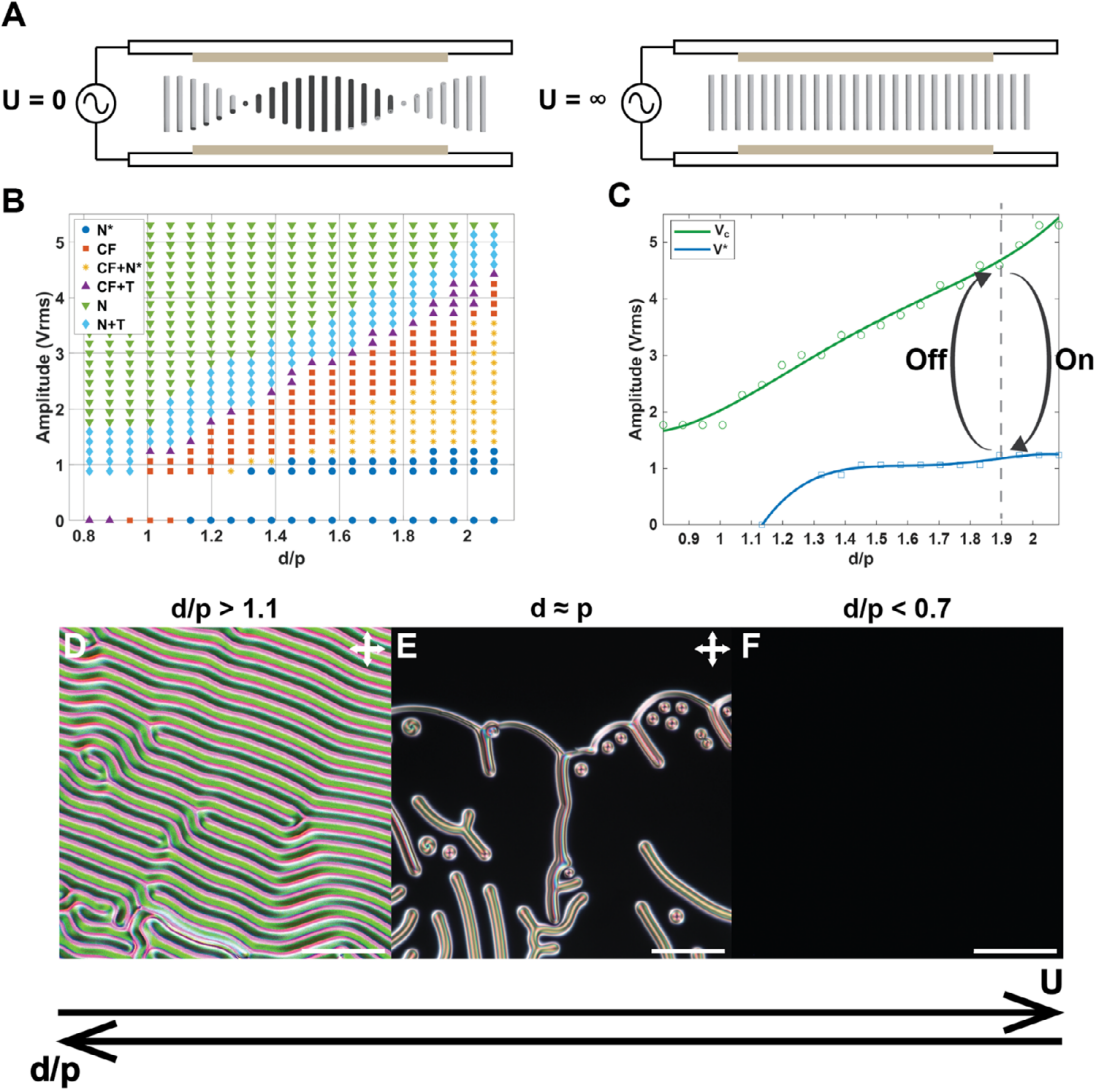
Electrical modulation of the effective pitch in homeotropic cells. (A) Scheme of the extreme case of cholesteric liquid crystal phase, completely wounded (left) in comparison to the fully unwounded state (right) by the application of an electric field onto a *Δε* >0 liquid crystalline media. (B) Stability diagram in the *d/p*, electrical potential space, of E7 with an initial pitch *p* = 12 µm, in a wedge cell with *d_min_* = 9 µm and *d_max_* = 25 µm. N*: Cholesteric phase with twisted structures, CF: Cholesteric Fingers, T: Torons, N: Unwound nematic‐like structural state. (C) Simplified version of (B), corresponding to the potential delimiting N* (U*) and N (U_c_), between those values, many solitonic structures coexist. (D–F) Polarized optical microscope images, of N* state (D) for *d/p* > *1.1*, CF+T state (E) for *d* ≂ *p*, and N state (F) for *d/p* < *0.7*. The scalebar corresponds to 100 µm and the direction of the crossed polarizers is shown in the inset.

The structural configurations of the cholesteric phase, as previously described [9, 10, 11], are highly sensitive to the ratio between the cell thickness (*d*) and the cholesteric pitch (*p*) under homeotropic boundary conditions [11, 12]. The two extreme cases are illustrated in Figure 1A.

On one hand, when d/*p* > 1.1, a continuously twisted helical structure forms along the substrate, resulting in a colorful, alternating pattern observed under a polarized optical microscope (POM) (Figure 1D). On the other hand, when *d/p* < *0.7*, the anchoring forces dominate the twisting tendency, leading to a uniform untwisted nematic‐like structure, which appears as a completely dark texture under POM (Figure 1F).

Between these two extremes, when *d*/*p* ≈ 1, a coexistence of the nematic‐like (N) unwound cholesteric and the twisted cholesteric (N*) structures emerges (Figure 1E). In this intermediate regime, the twist competes with the surface anchoring forces, giving rise to localized twisted regions surrounded by untwisted areas [9, 11].

These localized twists exhibit a variety of geometric forms. The worm‐like structures are known as cholesteric fingers (CF) [12], and they can coexist with circular‐shaped solitons commonly referred to as torons (T) [19] and various other chiral solitonic structures, including cholesteric finger loops (CFL) [24]. The dynamics, relaxation behavior, interaction with laser light, and potential applications of these structures are currently under investigation [17, 23, 24]. Moreover, various types of topological analogues of these structures are studied in chiral magnetic solids like iron germanium [25].

A wedge cell and an applied electric field were used to modulate and observe various textures in a positive‐dielectric‐anisotropy cholesteric liquid crystal, as summarized in the structural stability diagram (Figure 1B).

At *U* = 0, the wedge cell contains no unwound N regions. For *d/p* > 1.1, the texture is fully twisted (N*). Upon applying an electric potential, the onset of the cholesteric phase twisted structures (relative to its unwound nematic‐like N state) shifts toward the thicker part, gradually receding until it disappears entirely for *U* > 1.40 V_rms_. The unwound N state begins to appear in the thinnest part of the cell for *U* > 1.65 V_rms_, and the liquid crystal mixture can be completely unwound over the whole cell area for *U* ≈ 5.20 V_rms_.

This shift on the spatial distribution of N and N* is illustrated in Figure 1C, where the corresponding *U_d/p_* for N and N* have been respectively named *U_c_* and U*. Additionally, we observe the nucleation of torons in the vicinity of *U_c_*, as well as the spontaneous formation of cholesteric fingers between *U_c_* and U*, with a maximum density closer to U*.

### 2.2 Formation of Concentric Rings of Solitonic Cholestric Fingers

A first step in the formation of the ring structures is the local nucleation of torons or cholesteric finger loops (CFLs) by suddenly lowering the applied voltage just above the critical potential *U_c_*, after the liquid crystal texture has been fully unwound. From this voltage, switching to a second voltage *U_2_* (where *U_2_* < U*, black arrows Figure 1C) allows the twist to re‐emerge, leading to the formation of a continuous helix. This relaxation process occurs initially in the vicinity of pre‐existing twisted regions (Video S1).

**FIGURE 2:**
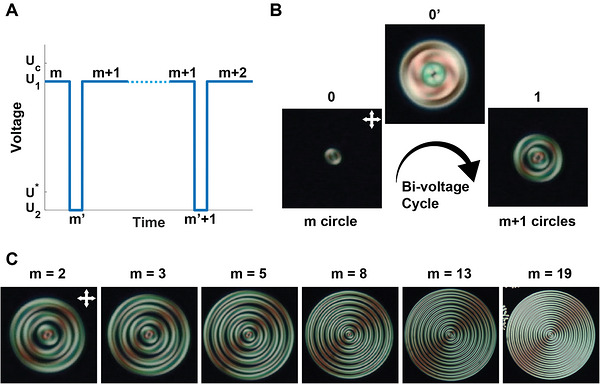
Generation of discrete cholesteric loops by a bi‐potential electric driving process. (A) Scheme of the bipotential process, qualitative representation of the electrical potential used, represented in Figure 1C by the black arrows. (B) Snapshot of Video S1 showing the first loop formation, obtained from the controlled relaxation of the chiral toron structure. (C) Snapshot of Video S1, for different numbers of concentric rings of solitonic cholesteric fingers. The width of ring CFL is approx. 5 µm. the structures diameter reaches ∼ 400 µm for *m* = 19.

If the transition to the twisted state N* is designed to be temporary by the use of a pulse‐like modulation of the potential (as illustrated in Figure 2A), we observe the formation of concentric rings around the initial toron or CFL (Figure 2B). This ring formation results from the discrete and localized reintroduction of the twist during a brief switch from *U_1_* to *U_2_*.

In Video S1, we observed the formation of multiple rings by repeatedly cycling the process, using a voltage driving scheme with *U_1_* = 3.64 V_rms_, *U_2_* = 1.08 V_rms_, and a switching duration *t* = 1.4 s at the wedge cell location corresponding to *d*/*p* = 1.9. With each cycle, a new ring was formed and stabilized (Figure 2C). The values for *U_1_*, *U_2_*, and *t* were determined empirically as optimal operating parameters, based on experimental observations, calibrations from the stability diagram (Figure 1B,C), as indicated by the black arrows, and the switching duration calibration (Figure S1).

For consistent pattern formation, *U_1_* must lie within the range where torons and CFLs are energetically stable or metastable, preventing their spontaneous collapse. *U_2_* must be close enough to *U_c_* to delay the bulk relaxation and kinetically favor twist formation around existing topological structures. The switching duration time *t* is also a key parameter that depends on the switching voltage *U_2_* (as observed in Figure S1).

On one hand, if *t* is too large, a bulk relaxation will occur, and a disordered pattern will form, losing the templating effect induced by the original topological solitonic structure that served as a “seed.” On the other hand, if *t* is too small, the minimum radius required for the next CFL will not be reached, preventing further ring formation.

Following the procedures stemming from the experimental insights, we achieved a topological structure composed of *m* = 19 solitonic finger rings, resulting in a pattern with a total diameter of ∼400 µm as observed in the snapshot of Figure 2C. This demonstrates that the process is both robust and reliable for patterning at large scale with pre‐designed topology. While we hypothesize that there may exist a theoretical limit to the maximum number of rings or overall structure size achievable with this method, such a limit was not observed in our experiments. The primary limitation encountered was the structural interference, as competing rings attempt to grow simultaneously (an effect visible in Video S2), though in future work it should be possible to pre‐select and control desired solitonic structures of this type.

### 2.3 Photopolymerization, Identification and Description of the Topological Structures

After understanding the formation mechanism of the concentric rings, we utilize the underlying director field to achieve an anisotropic mechanical response in the liquid crystal polymer coating. To enable this, we engineer a liquid crystal monomer mixture with positive dielectric anisotropy (*Δε* > 0) and a cholesteric phase temperature range that is experimentally accessible and easy to handle (Figure S2).

The compounds are summarized in the Figure S3 and consist of five reactive mesogens (RMs), with LC756 serving as the chiral dopant, combined to a photoinitiator that initiates radical polymerization upon UV illumination. This mixture is filled into a cell at *T*>*T_ni_* (nematic–isotropic transition temperature) with a thickness of *d *= 10 µm, yielding a ratio *d/p *= 2. Upon cooling to 55°C, this configuration results in the formation of the twisted N* structures.

**FIGURE 3:**
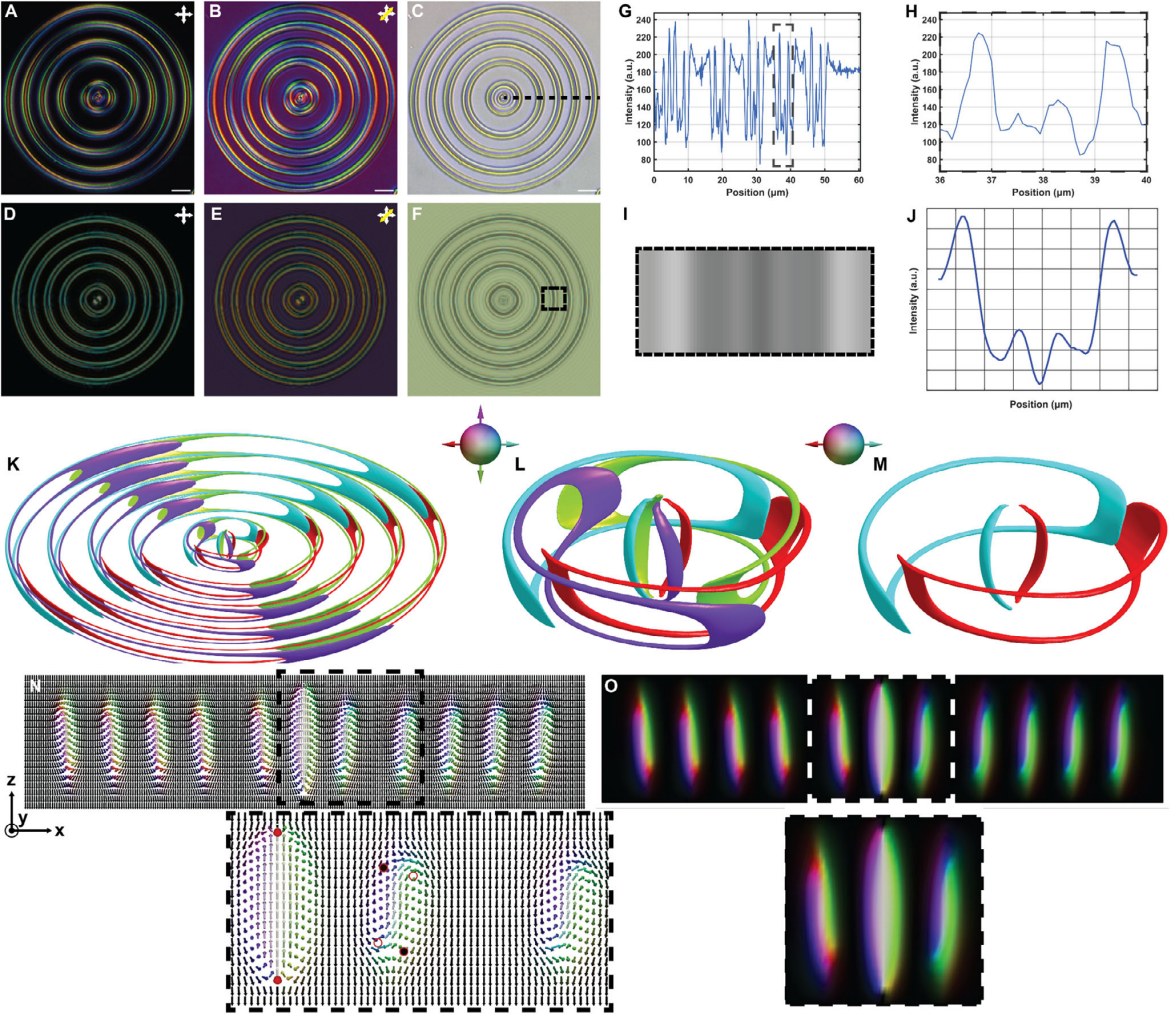
Structure and topology of the cholesteric solitons and solitonic cholesteric finger loops. (A) Polarizing optical microscope (POM) image of a topological structure composed of *m* = 5 rings with a central toron. (B) POM with an additional 530 nm wave plate depicted with the yellow double arrow. (C) Micrographs based on the observation using brightfield transmission‐mode optical microscopy. The dashed black line represented the intensity profile used for the CF type analysis. The scalebar corresponds to 10 µm. (D–F) Respective numerically simulated images based on the director field depicted in (K) using Nemaktis sofware with *d* = 10 µm, *n_e_* = 1.73, *n_o_* = 1.52. (G) Intensity profile taking along the radius of the structure with *m *= 5 circles observed using bright‐field microscopy (depicted with a dashed black line on (C). (H) Zoomed‐in profile of a cholesteric finger composing the fourth ring (depicted by the black dashed rectangle, (G). (I) Simulated brightfield microscopy of a CF1. (J) Corresponding intensity profile of a simulated CF1 structure.(K–M) Visualization of the director fields. The vectors decorating the nonpolar director via smooth vectorization, where they are colored based on their orientations as shown in the order‐parameter space (colored spheres). Preimages of the vectorized director orientation of the complete structure for four orientations (K), and zoomed‐in preimages to the core of the structure for four (L) and two orientations (M). (N) Cross section of the vectorized director field of a structure with *m* = 5, with a zoom and the toron and two other CF1Ls. (O) Cross section of the colored director field of a structure with *m *= 5, with a centered zoom of the toron and the CF1L.

After exploiting the phenomenon described above and using the following parameters [*U_1_* = 6.71 V_rms_; *U_2_* = 2.17 V_rms_ *; t* = 1.7 s], we photopolymerized the system by exposing the sample to UV light. Following the photopolymerization, the electric field was turned off, and the cell was mechanically opened. The POM images in Figure 3A–C show the preservation of a structure consisting of five concentric rings surrounding a central toron.

As discussed above, the rings are composed of cholesteric fingers (CFs). Four types of CFs have been reported, each characterized by distinct director field configurations and nucleation conditions [11]. To determine the nature of the observed CFLs, we followed two complementary identification approaches. The first is based on the comparison between simulated optical images (Figure 3D–F), which are numerically modeled based on computed energy‐minimizing director fields (Figure 3F,G), and experimental observations (Figure 3A–C). The second approach follows an approach from Oswald et al. [12], which establishes a clear correlation between CFs type and the light intensity distribution profile using brightfield microscopy (black dashed line in Figure 3C) for carefully selected samples. It shows two intense peaks at the edges of the structure combined with two peaks of different intensity closer to the center (Figure 3G–J). Based on both approaches, we conclude that the loops are composed of type‐1 CFs (CF1s).

A CF1 consists of a 2π twist along an axis tilted relative to the substrate plane, which is smoothly embedded in a uniform untwisted background. These smooth structures exhibit translational invariance along their propagation axis (i.e., along the loop) (Figure 3K–O), and their director field resembles a discretized version of the translationally invariant configuration [11], which reflects the nature of their formation process. By switching between two voltage states, the ring formation results from the discretization of the twist, driven by the tilt of the helical axis away from the cell normal. Additionally, each cholesteric finger contains four nonsingular disclination λ lines: two of charge −1/2 (dark) and two of charge +1/2 (white) highlighted by hollow spheres in Figure 3N. These nonsingular defect lines form a quadrupole of defect lines that arise while satisfying the homeotropic anchoring imposed by the boundary conditions.

The concentric rings are centered on a toron (Figure 3A,B) that served as a seed for the solitonic cholesteric finger loop formation; a generic form of such axisymmetric solitonic structure is often also referred to as a “cholesteric bubble” [26], albeit these “bubbles” historically referred to solely based on the quasi‐circular appearance may correspond to a plurality of topologically distinct structures such as skyrmions, skyrmion‐toron hybrids, hopfions, hopfion‐toron hybrids, fractional hopfions, etc [27, 28]. Since the topological structural aspects are important for the functionality, we analyze these objects based on the toron‐type topology rather than solely the quasi‐circular appearance under POM. At the mid‐plane, the toron is topologically equivalent to a two‐dimensional skyrmion [29]. In its simplest form, this centrosymmetric structure consists of a double twist of the director field with a skyrmionic topological nature, terminated near the substrates by two singular oppositely charged point defects required to fit the boundary conditions, as represented by the two spheres in Figure 3N.

The topology of observed configurations is analyzed with the help of displaying preimages of the single points on the order parameter space of the vectorized director field, the two‐sphere. The toron feature in the middle of the overall configuration has preimages of a single‐value vector field (and correspondingly colored points on the two‐sphere order parameter space) spanning between the singular point defects near the top and bottom surfaces (Figure 3K), consistent with it being a skyrmion tube terminating on two point defects. The solitonic cholesteric finger loops each feature closed‐loop preimages of distinct points on the two‐sphere order parameter space, albeit these loops corresponding to different points of the two‐sphere are unlinked with each other, reflecting the fact that the topology of these rings in terms of the third homotopy group is trivial (they are not hopfions). This is consistent with the observation that such finger CF1 loops can nucleate and disappear continuously out of the unwound background, which would be impossible for hopfions. However, topological features can be identified with fingers' cross‐sections (Figure 3N,O), as seen in the smoothly vectorized structures of the director field, where the so‐called “lambda line” disclinations (which are also referred to as fractional skyrmions and as “distwistinations” or “dischiraliations” in the context of their topology within the helical axis field [30] appear in pairs of opposite sign while forming a quadrupole within a CF‐1 cross‐section. The fact that the closed loops of preimages related to distinct director orientations are unlinked and the fractional skyrmions appear in self‐compensating pairs within the finger cross‐sections is instrumental for our dynamic generation of the concentric finger loops around torons without the emergence of singularities in the material director field.

### 2.4 Thermal Anisotropic Response of a Structure With *m* = 5

**FIGURE 4:**
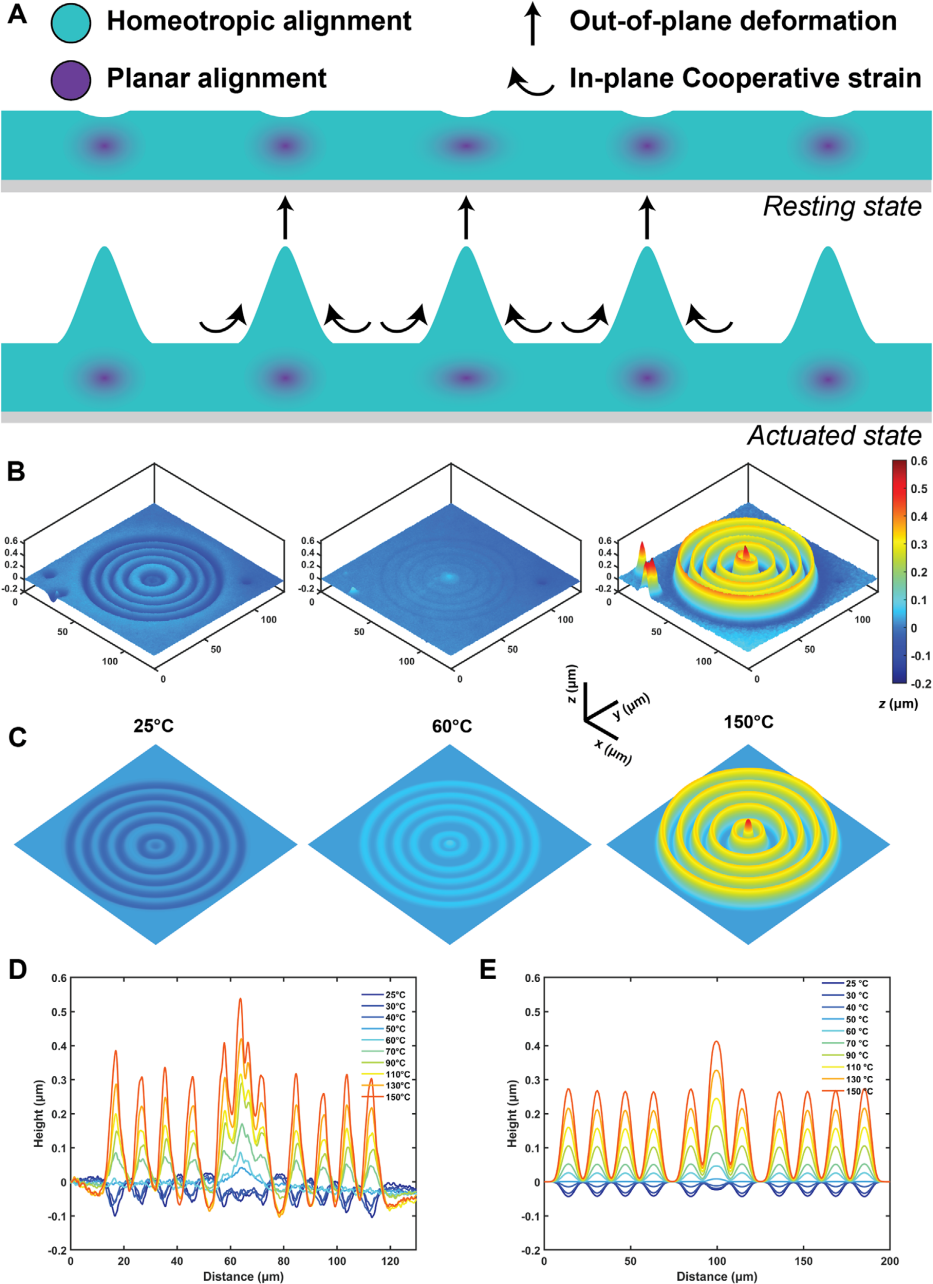
Actuation of polymerized homeotropically aligned liquid crystal with an embedded *m* = 5 topological structure. (A) Schematic of the thermally induced deformation mechanism associated with topological rings in a liquid crystal network. (B) Surface topography measured by white interferometry at 25, 60, and 150°C. (C) Corresponding simulated topographic deformation of the structure based on a voxel model combined with the computed director field. The colorbar (right‐side inset) applies to all surface plots. (D) Surface profiles across the structure at different temperatures. (E) Corresponding simulated profiles based on the voxel model (supplementary information) [20].

After an initial heat treatment to relieve stress induced by the polymerization process, white light interferometry reveals the formation of surface indentations patterned by the previously described structure. A central indentation of approximately 60 nm is surrounded by five concentric indented rings (*m* = 5) of similar depth (Figure 4B, left panel). The lateral dimensions of these indentations correspond to the spatial extent of the torons and CF1L structures, respectively. The formation mechanism of these indentations has been described elsewhere [20]. Briefly, polymerization was carried out at a temperature above room temperature, effectively “freezing” (solidifying) the system with an order parameter slightly lower than that at room temperature. Upon subsequent heat treatment and relaxation back to room temperature, the order parameter slightly increases, leading to shrinkage‐induced stress in regions where the director is oriented in‐plane (relative to the substrate), and expansion‐induced stress in homeotropic regions, where the director is aligned orthogonally to the surface. When the system is heated again, the inverse occurs due to a restricted decrease in the order parameter (Figure 4A).

Near the polymerization temperature (*T* = 60°C), a quasi‐flat state is observed (Figure 4B, mid panel). Above this threshold, the ring structures elevate, forming at 150°C a series of concentric walls approximately 350 nm high, with spacing corresponding to the ∼10 µm distance between adjacent CF1L features (Figure 4B, right panel). The central peak rises to ∼500 nm, forming a volcano‐like tip characteristic of surfaces templated by torons, resulting in an overall castle‐like morphology (Figure 4D). These experimental observations align well with results of our computational modeling (Figure 4C,E). In this model, the equilibrium director field is derived from a minimization of the Frank‐Oseen free energy on a large grid, and an effective height is assigned to each voxel using an empirically fitted height function, as previously reported [20].

### 2.5 Library of Structure up to *m* = 5

**FIGURE 5:**
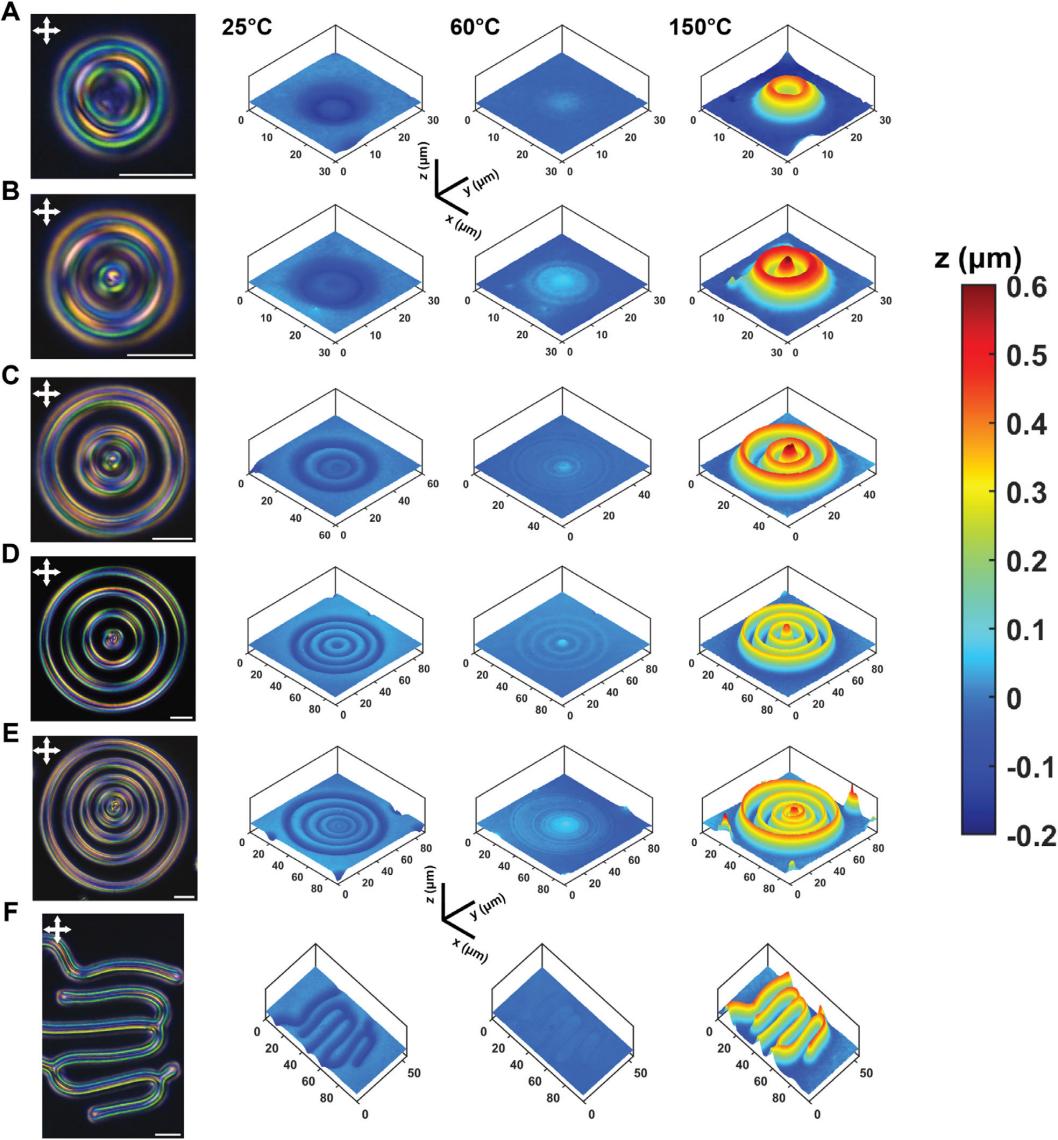
An assortment of structures and their corresponding thermal actuation. (A–F) POM images (Left panel) and surface at different temperatures (25, 60, and 150°C) of a single CF1L without toron (A). A structure with *m* = 1 (B), *m* = 2 (C), *m* = 3 (D), *m* = 4 (E) and a duplicated CF1 line forming an S‐shaped structures. The colorbar applies to all surface plots. The scalebar corresponds to 100 µm and the directions of the crossed polarizers are shown by white double arrows.

By using this process, structures with a predetermined number of concentric finger loops could be achieved. Moreover, due to the stepwise relaxation process of the rings (which occurs through the center, as seen in Video S3), structures without the centered toron can be obtained. In Figure 5A, an example is shown featuring a single CF1L with a black central region, resulting in the inversion of the initial indentation upon thermal stimulus. This leads to a volcano‐like deformation, reaching a maximum height of approximately 600 nm at 150°C, along with a 200 nm indentation at the tip. Furthermore, the tunability of the central region, as well as the size and number of finger CF1 loops in the castle‐like structure, can be controlled by selecting the number of rings, as demonstrated in Figure 5B–E for *m* = 1 to 4. Overall, the deformation amplitude is similar across these structures, as it primarily depends on the nature of the nonsingular structures that compose them. The profiles at different temperatures for all those structures are exhibited in Figure S4.

In addition to circular‐type of structures, in Figure 5F, an S‐like structure composed of CF1 is shown and obtained by the same methods previously discussed by using a CF1 line as a starting point. This is to illustrate that the process replicates the initial conditions (As illustrated in Video S4), and many configurations could substantially be used as a template. In this case, it results in S shape surface that behaves in the same way to thermal actuation, with an inversion occurring a polymerization temperature. Additionally, these structures retain their birefringent properties across all tested temperatures (Figure S5) and demonstrate robust thermal stability over 25 heating–cooling cycles (Figure S6).

**FIGURE 6:**
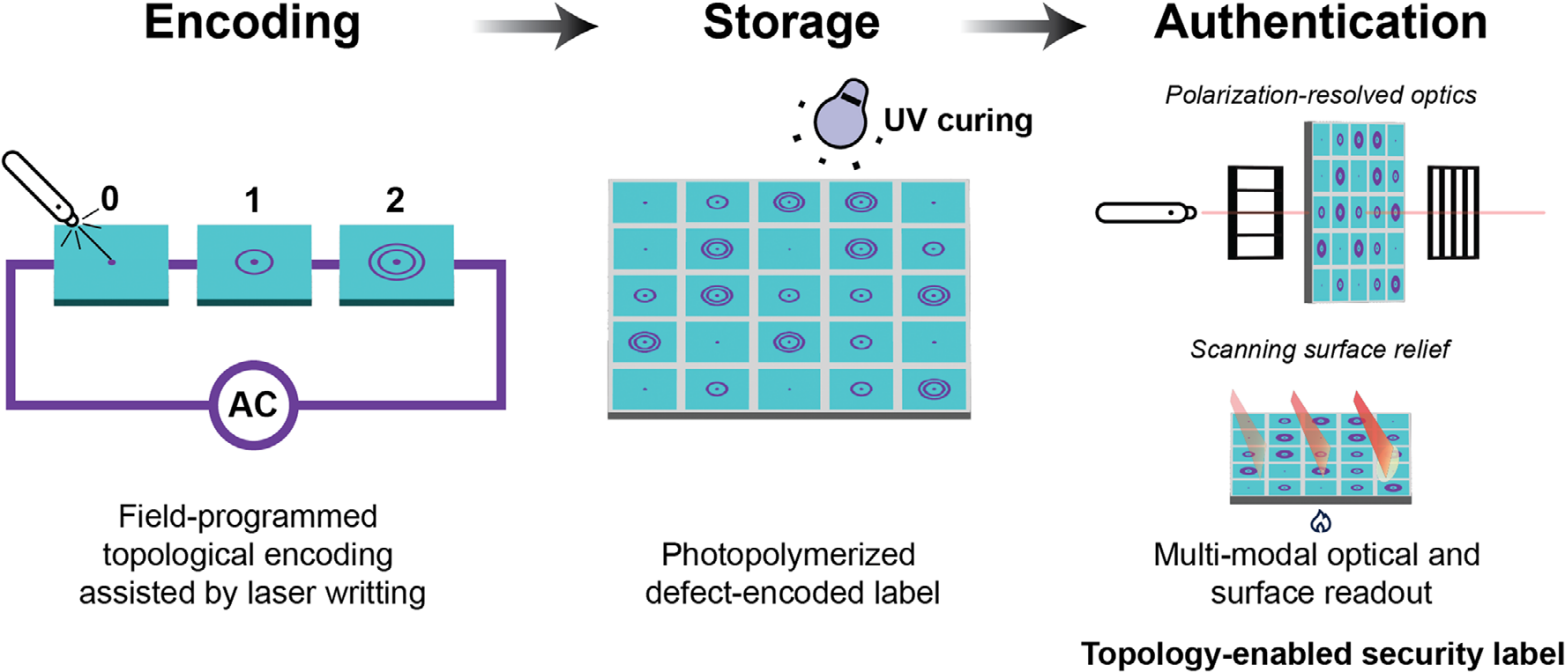
Conceptual framework for topology‐enabled multi‐modal security labels for anticounterfeinting applications. Field‐programmed topological structures are generated through electrically assisted laser writing (Encoding) and subsequently fixed by photopolymerization (Storage). The resulting encoded patterns can be read out via polarization‐resolved optics or thermally induced surface relief (Authentication), enabling multi‐modal and robust anti‐counterfeiting strategies.

The combination of thermal stability, retention of birefringent properties across different temperatures, and the large library of generated structures with tunable surface morphologies makes these systems promising candidates for multi‐modal, topology‐enabled security labels, as conceptually illustrated in Figure 6.

## 3 Discussion and Conclusions

We have demonstrated the potential of an innovative approach that leverages the complexity and metastability of torons and cholesteric fingers, solitonic topological structures in chiral liquid crystals. Using a bi‐potential electric cycling process, we achieved precise and reliable regeneration of initial topological solitons from a toron‐like topological seed, enabling the combination of cholesteric‐pitch‐scale resolution with large‐scale patterning of photo‐ and thermally‐responsive coatings. This process results in a topological structure composed of *m* concentric rings, where *m* corresponds to the number of applied electrical cycles. These rings consist of type‐1 cholesteric finger loops surrounding a central toron.

The resulting topological structures were embedded and stabilized within a unidirectionally aligned liquid crystal network using a reactive mesogen mixture specifically formulated for this purpose. Following UV photopolymerization, the structures remained fully preserved even after turning off the electric field. Their distinct and complex configurations enable reversible surface topography modulation: initially forming ripple‐like structures at room temperature, with indentations corresponding to the position of the spatially localized twisted solitonic regions. Upon heating to 150°C, these morphologies transition into castle‐like structures, with the inversion occurring at the temperature of polymerization. The surface height change across all structures is consistently around ∼700 nm. All observations are supported by our numerical model.

We expect the method described in this work to be applicable to a wide range of materials with a positive dielectric constant. Furthermore, the process could be enhanced by integration with established laser‐writing techniques [19], which would enable precise spatial control over the formation of cholesteric finger loops and torons as conceptually illustrated in Figure 6. In addition, laser writing could serve as an erasing tool to eliminate interference between neighboring structures, as demonstrated in this work. We believe that CF1Ls and torons are only two examples within a broader class of topological solitons whose relaxation dynamics can be harnessed for large‐scale templating. The exotic director field configurations of these structures give rise to strong optical anisotropy and dynamic, reversible surface morphologies. These features make them promising candidates for tunable optical elements, particularly through spatial engineering of the optical path length, which could lead to a new generation of meta‐optics devices [31, 32, 33]. The emergence of well‐defined stimuli‐responsive topography could potentially find utility in anticounterfeiting applications [34]. Beyond optics and anticounterfeiting, potential applications may extend to soft robotics [35], haptics [36], chemical sensing [37], and molecular trapping [38].

## 4 Experimental Section/Methods

### 4.1 Materials

The liquid crystal mixture was composed of six components, as detailed in Figure S2 (Supporting Information). Components **1–4** were monomers obtained from Merck UK. Component **5** was a chiral monomer containing an asymmetric carbon center, and component **6** was a photoinitiator (Irgacure 819), both obtained from BASF. The proportions of each compound used in the formulation are summarized in Table S1.

### 4.2 Substrate Preparation

The glass substrates covered with ITO were cleaned by two sonication baths: first with acetone, followed by propanol‐2 for 20 min each. The surface was activated by ozone exposure (UV–ozone photoreactor PR100) for 20 min. The glass slides were spin‐coated (using a commercial instrument LaurellWS‐650‐23) with a polyimide layer inducing homeotropic alignment (SE4811 Nissan SUNEVER) (STEP1: 800/5/500 STEP2: 5000 rpm /40s/800 rpm s^−1^). Subsequently, they were prebaked at 90°C for 10 min and baked at 190°C for 90 min. The cells were prepared by using glue containing 1w/w% of bead spacers with diameters depending on the thickness required.

### 4.3 Wedge Cells

The cells were composed of glass covered with ITO assembled using two different spacers (6/25 µm), and the resulting wedge angle was verified using Bruker Dektak XT (With a 2 µm radius tip) and the thicker part used as a reference point. The wedge cell used in the stability diagram (Figure 1B,C) had a length of 2 cm, *d_min_* = 9 um, and *d_max_* = 25 µm. The composition used was a mixture of E7 with LC756 designed to obtain a pitch *p *= 12 µm and inserted by capillary forces at a temperature above *T_ni_*.

### 4.4 Sample Preparation

The cells were filled at a temperature *T*>*T_ni_* and slowly cooled down to room temperature for E7 and to 55°C for the reactive mixture. After obtention of the desired pattern, the liquid crystalline network was formed by photopolymerization by exposure to a 365 nm LED and the corresponding LED controller (DC4104, Thorlabs) set at a power of 100 MW/cm^2^ at 55°C. Following polymerization, the cell was mechanically opened and subjected to an initial thermal treatment at 100°C for 5 min. This step was performed to release internal stresses induced during the polymerization process.

### 4.5 Electrical Field Application

The alternating electric field was provided by a function generator (AFG3252C, Tektronix). The electric signal from the function generator was amplified through a high‐voltage linear amplifier (WMA‐300, Falco Systems). Generation of arbitrary waves was done using MATLAB and imported to the function generator using the ArbExpress software. A sinusoidal wave of frequency 1 kHz was used for the E7 mixture. An envelope‐modulated signal with a carrier frequency of 1 kHz and an envelope of 50 Hz was used for the generation of structure in the polymerizable mix. The applied voltage was controlled by an in‐house MATLAB script.

### 4.6 Surface Characterization and Actuation

The thickness of the coating and surface modulation were recorded using white light interferometry (Sensofar Neox) coupled to a hotplate (Linkam TMS 94). To ensure full relaxation of the coating at the desired temperature, a waiting time of at least 30s is employed before any acquisition. Topological soliton recognition, analysis, and localization were done by using a polarized optical microscope (Leica DM6000M).

### 4.7 Analysis and Optical Characterization of Topological Structures

Topological defect recognition, analysis, live observation of the phenomena, and localization have been done using a Polarized Optical Microscope (DM6000M, Leica) equipped with a CCD camera (DFC 420 C, Leica).

Differential Scanning Calorimetry: The thermal transition point of the different mixtures was determined via differential scanning calorimetry (DSC) using a TA instrument (Q2000, DSC). DSC measurements consisting of three repeating cycles were performed from −50 to 150°C at 10°C min^−1^.

### 4.8 Director Structure Simulation

All relaxed free‐energy‐minimizing structures of the chiral nematic before polymerization were modeled by numerically minimizing the Frank–Oseen free energy to an equilibrium state on a discretized 3D grid [39]. Further information on modeling is available in the Supporting Information.

### 4.9 Optical Image Simulation

Each image was computed via the software Nemaktis [40] by importing the corresponding relaxed structure, cell dimensions, and refractive indices. The refractive indices used for the simulated POM images were measured previously in a similar system [41]. More information on this modeling is available in the Supporting Information.

### 4.10 Topography Actuation Modeling

The surface topography was obtained from each relaxed structure by assigning an effective height function to each column oriented along the z‐axis and summing over the effective heights to compute the overall topography. See the Supporting Information and previous publication [20] for more details.

### 4.11 Machine‐Generated Content

AI‐assisted tools were used solely for language editing and structural refinement. All scientific content was created, reviewed, and verified by the authors.

## Author Contributions

**Jacques A. Peixoto**: conceptualization, investigation, writing – original draft, methodology, writing – review and editing, visualization, software, validation, formal analysis, resources. **Dirk J. Broer**: supervision, writing – review and editing, methodology. **Hanqing Zhao**: validation, software, formal analysis, visualization, writing – review and editing, resources. **Ivan I. Smalyukh**: supervision, validation, writing – review and editing, project administration, funding acquisition, methodology. **Danqing Liu**: supervision, writing – review and editing, funding acquisition, project administration, resources, methodology.

## Conflicts of Interest

The authors declare no conflicts of interest.

## Supporting information




**Supporting File 1**: advs76885‐sup‐0001‐SuppMat.docx.

**Supporting File 2**: advs76885‐sup‐0002‐VideoS1‐S4.zip.

## Data Availability

The data that support the findings of this study are available from the corresponding author upon reasonable request.
