# Supplementary material for: Thermal Control of Concentric Topographies Patterned by Dynamic Electro‐Templated Generated Chiral Solitonic Structures
Source: Adv Sci (Weinh). 2026 Jul 29;13(60):e76885. doi: 10.1002/advs.76885 (PMC13418053; doi:10.1002/advs.76885)
Supplement: Supplementary file 1 [file ADVS-13-e76885-s002.docx]

Supporting Information

Thermal control of concentric topographies patterned by dynamic electro-templated generated chiral solitonic structures

Jacques A. Peixoto, Hanqing Zhao, Dirk J. Broer, Ivan I. Smalyukh*, and Danqing Liu*.

**Approaches and implementation of numerical modeling.**

To obtain equilibrium or metastable structures of solitons like torons and cholesteric fingers of the first type, we relax the structures from an ansatz on a discretized 3D grid representing the director field ***n****(****r****)* by numerically minimizing the Frank-Oseen free energy

$$\begin{aligned} F=\int d^{3}x\left[ \frac{K_{11}}{2}\left( \nabla\cdot n \right)^{2}+\frac{K_{22}}{2}\left( n\cdot\left( \nabla\times n \right)+q \right)^{2}+\frac{K_{33}}{2}{\left( n\times(\nabla\times n \right))}^{2} \right]\#\left( 1 \right) \end{aligned}$$

as described in detail in previous articles^[1,2]^, where $K_{11}$, $K_{22}$, and $K_{33}$ are the Frank-Oseen elastic constants, assigning an energetic cost for splay, twist, and bend deformations, respectively. The parameter $q=2\pi/p$ is the chiral wavevector and $p$ is the cholesteric pitch, with both $p$ and $q$ describing the tendency of chiral liquid crystals to twist while achieving lower free energy. We relax each structure by iteratively solving the Euler-Lagrange equations for Equation S1 with the finite difference method. To model samples with voltage application, an additional term is added to the free energy to describe director’s coupling with the electric field ***E***,

$$\begin{aligned} F_{E}=-\frac{\epsilon_{0}\Delta\epsilon}{2}\int d^{3}x\left( \boldsymbol{n\cdot E} \right)^{2}\#\left( 2 \right) \end{aligned}$$

where $\boldsymbol{E}$ is the applied electric field, $\Delta\epsilon$ is the dielectric anisotropy, and $\epsilon_{0}$ is the permittivity of free space. Director field preimages were obtained from simulations by the condition $\left| \boldsymbol{n}\left( \boldsymbol{r} \right)-\boldsymbol{n}_{0} \right|<\delta$, which produces a preimage of the vectorized director $\boldsymbol{n}_{0}$ with tolerance $\delta$. For illustrative purposes, we selected the tolerance $\delta=0.3$.

The computer-simulated POM images for the textures in this paper were generated via the open-source Nemaktis software^[3,4]^ implementation of the diffractive transfer matrix method, where the cell thickness is 10 μm, ordinary refractive index is 1.52 and extraordinary refractive index is 1.73.

In our modeling of surface topography, the height actuation is extracted by assigning each voxel an effective height,

$$\begin{aligned} h\left( \theta,T \right)=\sqrt{\left( h_{||}\left( T \right)cos \left( \theta\right) \right)^{2}+\left( h_{\perp}\left( T \right)\sin\left( \theta\right) \right)^{2}}\# \end{aligned}(3)$$

along the z-axis. Here $\theta$ is the local polar angle that the director makes relative to the homeotropic background (along the cell normal) and $h_{\parallel}$, $h_{\perp}$ represent the homeotropic and planar expansion coefficients at temperature *T*. Further details could be found on our previous publication^[5]^.

Figure S1 Evolution of the pulse duration required to generate a loop as a function of the applied voltage. The minimum and maximum times correspond to the shortest time required to form a cholesteric loop and to the complete reconfiguration of the system, respectively. Symbols represent experimental data, while dashed lines indicate polynomial fits.

Figure S2 DSC diagram of the polymerizable mixture composition showing a melting temperature at 52.0 ^o^C, and a chiral-nematic transition to the isotropic phase at 64.9 ^o^C. During cooling the material can still be processed in its monotropic supercooled state

Figure S3. List of compounds composing the polymerizable mixture. The monomers are selected to provide a Δε>0 and optimized for processability. 1-2 are reactive mesogen with di-acrilate functionalization. 3-4 are reactive mesogen with mono-acrylate functionalization. 5 is LC756 used as chiral dopant with an HTP = +56 µm-1 [5]. 6 is the photoinitiator.

Figure S4. (A-F) Surface profiles at different temperatures, obtained by white light interferometry, correspond to the structures shown in Figure 5. The profiles are taken along the diameters of the overall structures.

Figure S5. Polarized optical microscopy images of a structure with m = 1 at different temperatures (25, 100, 150, and back to 25 °C). The scale bar corresponds to 20 µm. The crossed polarizers are indicated by white arrows.

Figure S6. Polarized optical microscopy images of a structure with m = 1 after different heating–cooling cycles (25–150 °C). The scale bar corresponds to 20 µm. The crossed polarizers are indicated by white arrows.

*Table S1. Exact composition used for the polymerizable mixtures. The compounds numbered are the one from Figure S3. The mixture has been made to achieve a pitch equal to 5 µm with an expected HTP = 63 µm^-1^.*

| Compound | **1** | **2** | **3** | **4** | **5** | **6** |
| --- | --- | --- | --- | --- | --- | --- |
| w/w% | 16.671 | 12.671 | 29.75 | 39.75 | 0.1587 | 1 |

**Supplementary Videos:**

Video S1. Generation of multiple rings using the process described in Figure 1 and Figure 2. Playback speed: ×10.

Video S2. Generation of rings in contact with other structures, resulting in an incomplete process. Real-time observations.

Video S3. Mechanism of relaxation at a defined voltage U > U_c_. Playback speed: ×20.

Video S4. Duplication of a double ring when sufficient space is available inside the structure. Real-time observations.

**References**

[1]	J.-S. B. Tai, I. I. Smalyukh, *Phys. Rev. E* **2020**, *101*, 042702.

[2]	G. Durey, H. R. O. Sohn, P. J. Ackerman, E. Brasselet, I. I. Smalyukh, T. Lopez-Leon, *Soft Matter* **2020**, *16*, 2669.

[3]	G. Poy, S. Žumer, *Soft Matter* **2019**, *15*, 3659.

[4]	G. Poy, S. Žumer, *Opt. Express, OE* **2020**, *28*, 24327.

[5]	J. Peixoto, D. Hall, D. J. Broer, I. I. Smalyukh, D. Liu, *Advanced Materials* **2024**, *36*, 2308425.
